# Editorial Note: PHYTOCHROME C regulation of photoperiodic flowering via *PHOTOPERIOD1* is mediated by *EARLY FLOWERING 3* in *Brachypodium distachyon*

**DOI:** 10.1371/journal.pgen.1011592

**Published:** 2025-02-27

**Authors:** The PLOS Genetics Editors

This Editorial Note is issued to provide an update to the Expression of Concern notice previously issued on this article [1, 2].

At the time the interim Expression of Concern notice was issued [2], the authors were in the process of repeating the experiments underlying the results presented in the original Fig 2 (S2 File). Due to the time needed to conduct and review these experiments, the replication data were not included with the Expression of Concern notice [2] and are being provided in this follow-up notice (S1 File). Information on the repeat experiments and how these data affect the published results is available with this notice. The results of repeat experiments were assessed by a reviewer of the original article, who concluded that the repeat experiment data are consistent with the original study and that the overall conclusions stand.

Specifically, the authors repeated the gene expression level analyses and plant flowering and leaf number phenotypes that were presented in the original Figs 1 and 2 (S2 File) using samples obtained from new plant materials. The authors clarified that there was not sufficient leftover seed of the lot of *elf3/phyC* double mutant seed used in the original study, and therefore a new batch of *elf3/phyC* double mutant seed was generated by crossing single mutant plants containing the alleles of *elf3* and *phyC* used in [1]. Furthermore, the authors state that plants were grown in a reach-in, fluorescent-bulb-illuminated chamber with environmental conditions similar to those used for Fig 6 (S2 File) and that four biological replicates, with two leaves per replicate, were harvested for each genotype, and newly formed fourth leaves that had just fully expanded were chosen for analysis. The authors also clarify that the plants used for the results presented in the original Fig 2 (S2 File) were grown in a chamber illuminated with a combination of high-pressure sodium and metal halide lamps, whereas in these repeated experiment the plants were grown under fluorescent lights because chambers with high-pressure sodium and metal halide lamps were not available at the location where the experiments were repeated. The supporting data for the modified Figs 1 and 2, and the added Fig 3, are provided in S1 File.

The authors state that the major conclusions reported in the original study remain unaffected, but that the replicate experiments highlighted some differences in the expression results reported in the originally published Figs 1 and 2 (S2 File). In addition, differences were observed in the flowering time of Bd21-3, *elf3*, *phyC*, and *elf3/phyC* double mutant plants but are consistent with what was previously published. Furthermore, the authors state that in their efforts to repeat the experiments in the originally published version of Fig 2 (S2 File) they also analyzed gene expression in plants from the developmentally later 5th leaf stage and found *FT1* and *VRN1* are elevated in the *elf3/phyC* double mutant compared to wild-type and the *phyC* single mutant as reported in the original paper (S2 File). They provided an additional figure, the new Fig 3, to communicate the results of this additional experiment.

In light of these difference this article [1] was republished on January 24, 2025, to implement the following changes:

- Figs 1 and 2 have been updated in line with the repeat experiment results. The figure legends for Figs 1 and 2 have been updated.
- A new Fig 3 has been added. As a result, the original Figs 3–6 have been updated to Figs 4–7.
- The second paragraph of the results subsections **Rapid flowering of *elf3* is epistatic to the delayed flowering of *phyC*** has been updated.
- Both paragraphs of the Results subsection **Effect of mutations in *PHYC* and *ELF3* on the transcriptional profiles of flowering time genes** have been updated.
- The paragraph titled **The Effect of *PHYC* and *ELF3* on Days to Heading and the Expression of Genes Controlling Flowering Time** has been added to the discussion section and a description of the differences in plant growth conditions in experiments done at the University of California-Davis compared to the University of Wisconsin-Madison that may have led to the observed differences in the flowering time of wild type and various mutant lines are presented in the methods section.

Please download this article again to view the correct version. The original version of the published article remains available in the S2 File provided with this notice. The S3 File highlights the changes made to the article’s text at the time of republication.

The PLOS Publication Ethics team reviewed this case in collaboration with the *PLOS Genetics* Editors, and carefully considered case details including the confirmed data manipulation, the observation that the data in question comprise a relatively minor portion of the results, and the reliability of the article’s main findings as demonstrated by the repeat data and supported by an Editorial Board member. PLOS issues this Editorial Note to inform readers that the Expression of Concern [2], issued previously to inform readers of the data manipulation concerns and that findings in the originally published Fig 2 are unreliable, is final. However, the journal stands by the article once amended with this notice to include these alerts and the data obtained in repeat experiments.

## Supporting information

S1 File Underlying data for updated Figs 1–2 and new Fig 3. (XLSX)

S2 File Originally published PDF of this article [1]. (PDF)

S3 File Manuscript with tracked changes to highlight updates to results and discussion section following repeat experiments. (DOCX)
